# Novel Application of STRATOS for Restoration of Clavicular Stability After Oncologic Sternoclavicular Joint Resection: A Case Report and Review of the Literature

**DOI:** 10.3390/jcm15052002

**Published:** 2026-03-05

**Authors:** Shehab Mohamed, Luca Bertolaccini, Roberto Gasparri, Giorgio Lo Iacono, Antonio Mazzella, Monica Casiraghi, Claudia Bardoni, Cristina Diotti, Lorenzo Spaggiari

**Affiliations:** 1 Department of Thoracic Surgery, IEO—Istituto Europeo di Oncologia, European Institute of Oncology IRCCS, 20141 Milan, Italy; luca.bertolaccini@unimi.it (L.B.); giorgio.loiacono@ieo.it (G.L.I.);; 2 Department of Oncology and Hemato-Oncology, University of Milan, 20122 Milan, Italy

**Keywords:** chondrosarcoma, STRATOS bar, chest wall reconstruction

## Abstract

Background. Chondrosarcomas of the manubrium are exceedingly rare, accounting for approximately 20% of all primary bone malignancies, and present unique challenges in surgical management and reconstruction. Reliable reconstructive strategies for medial clavicular stabilization remain limited. Case Presentation. We report the first documented use of STRATOS bars for unilateral clavicular stabilization following manubrial chondrosarcoma resection. A 19-year-old woman with a poorly differentiated (G3) chondrosarcoma of the manubrium underwent neoadjuvant chemotherapy followed by en bloc resection of the manubrium, medial clavicle, and first rib. Reconstruction and clavicular stabilization were achieved using STRATOS, which is traditionally employed for chest-wall reconstruction. This represents a novel use of the device for medial clavicular stabilization after SCJ resection. At the 6-month follow-up, the patient remained disease-free, with preserved shoulder function and stable reconstruction. STRATOS provided stable fixation, preserved shoulder function, and an excellent cosmetic outcome. A brief review of the literature on sternal chondrosarcoma and reconstruction techniques is also presented. Conclusion. This unique application expands the reconstructive possibilities of modular titanium systems. It may offer a more reliable biomechanical alternative to traditional fixation methods in cases requiring stability of the shoulder girdle after SCJ resection. Further validation through biomechanical studies and larger case series is warranted.

## 1. Introduction

Chondrosarcoma is the second-most common primary malignant bone tumor after osteosarcoma, accounting for approximately 20% of all primary bone malignancies [1]. It most frequently arises in the pelvis, proximal femur, and humerus, whereas sternal involvement, particularly of the manubrium, is exceptionally uncommon, representing less than 2% of all chondrosarcomas [2,3]. Sternal chondrosarcomas typically present as slowly enlarging, painless anterior chest-wall masses and exhibit locally aggressive behavior with the progressive invasion of adjacent soft tissue and costal cartilage. Despite their locally infiltrative nature, distant metastases are relatively rare, accounting for 8–38% of chondrosarcoma patients, and generally occur late in the disease course [4,5]. Because chondrosarcomas are largely resistant to chemotherapy and radiotherapy, wide en bloc resection with negative margins remains the cornerstone of curative treatment [6,7,8]. Surgical management of tumors involving the manubrium, however, presents a unique challenge: the need to achieve oncologically sufficient margins while maintaining the mechanical stability of the sternoclavicular junction (SCJ) and the upper thoracic cage. The loss of the SCJ can lead to shoulder girdle instability, functional limitations, and poor aesthetic outcomes unless a rigid, anatomically appropriate reconstruction is achieved [9,10]. A variety of reconstructive strategies have been described, including autologous bone grafts, titanium plates, and composite meshes, each with variable outcomes regarding stability and motion preservation [10,11]. More recently, titanium modular systems such as STRATOS (Strasbourg Thoracic Osteosynthesis System) have been introduced for chest-wall stabilization following trauma, infection, or tumor resection [12,13,14]. This system consists of precontoured titanium bars and rib clips that can be adapted to the patient’s anatomy, providing stable, lightweight fixation with compatibility for postoperative imaging. While STRATOS bars have been successfully used for sternal pseudoarthrosis, post-transplant sternal dehiscence, and extensive chest-wall reconstructions [12,13,14,15], their application for medial clavicular fixation following SCJ resection remains largely unexplored. However, the biomechanical demands placed on the clavicular–sternal junction following oncologic resection remain insufficiently addressed by currently available reconstructive solutions. There is a lack of standardized approaches capable of providing both rigid fixation and the preservation of physiologic shoulder biomechanics, particularly in young and active patients. The clavicle behaves mechanically as a curved elastic beam that transfers loads from the upper limb to the axial skeleton. Under physiological conditions, bending moments generated by the weight of the upper limb and scapulothoracic motion can be approximated using the relation M=F·d, where *F* represents the resultant muscular and gravitational forces and *d* the perpendicular distance from the clavicular axis. Following sternoclavicular joint resection, this natural load path is disrupted, producing abnormal shear and torque forces at the clavicular stump. STRATOS restores this pathway by functioning as a structural beam whose flexural rigidity is given by EI, where E denotes the Young’s modulus of titanium and *I* denotes the second moment of area. This mechanical behavior allows the reconstructed construct to approximate the physiological stress–strain conditions of the native clavicle. Hajjar et al. [16] first described the use of STRATOS bars for bilateral clavicular stabilization after manubrial resection in a patient with thyroid carcinoma, demonstrating preserved shoulder function and excellent cosmetic outcomes. Building upon this experience, the present case describes a novel adaptation of STRATOS for unilateral clavicular stabilization after the resection of a high-grade manubrial chondrosarcoma, achieving satisfactory biomechanical and functional results. This case report details the first documented application of the STRATOS bar for unilateral clavicular stabilization after manubrial chondrosarcoma resection, emphasizing its biomechanical feasibility and its capacity to restore the functional alignment of the shoulder girdle. This report also contextualizes the technique within the existing literature on chest-wall and sternoclavicular reconstruction.

## 2. Case Presentation

**Figure 1:**
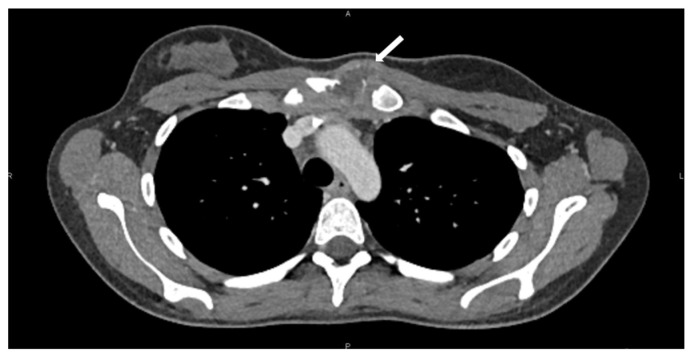
Preoperative CT scan showing the lesion (white arrow) involving the left hemimanubrium, medial clavicle, and first rib.

A 19-year-old woman presented with progressive pain and swelling over the left SCJ following minor upper extremity trauma. CT imaging of the neck and chest revealed a 39 × 28 mm lesion involving the left hemimanubrium, medial clavicle, and first costal cartilage with soft tissue extension. PET-CT demonstrated intense uptake (SUVmax 7). A CT-guided biopsy confirmed a poorly differentiated (G3) chondrosarcoma. No distant metastases were identified (Figure 1). The patient received six cycles of neoadjuvant chemotherapy consisting of four cycles of high-dose methotrexate and two cycles of cisplatin (CDP)-docorubicin (ADM), which resulted in the radiological stability of the lesion. Restaging imaging showed that the tumor remained localized to the manubrium with limited invasion of adjacent structures, and the case was discussed on a multidisciplinary tumor board. Surgical resection was then planned. The decision to proceed with surgery rather than additional systemic therapy was based on multidisciplinary consensus and the limited expected benefit of further cytotoxic treatment in high-grade chondrosarcoma. The lesions’ location and involvement of the SCJ required preoperative planning that was equally focused on oncologic clearance and the restoration of clavicular stability.

1. Operative Technique

**Figure 2:**
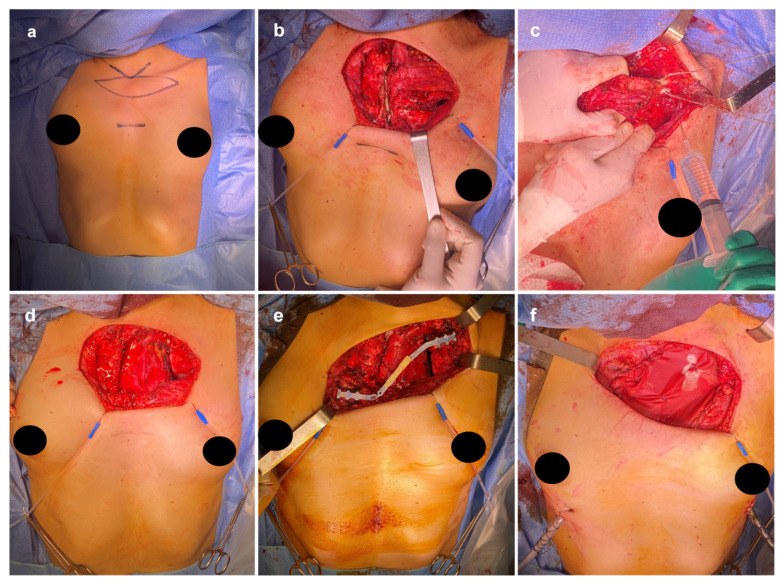
Intraoperative surgical steps for operative technique, reconstruction, and stabilization. (**a**) Preoperative marking of the planned surgical incision overlying the palpable anterior chest-wall lesion. (**b**) Exposure of the tumor through the initial skin and subcutaneous dissection, demonstrating involvement of the left hemimanubrium and medial clavicle. (**c**) Resection of the medial third of the left clavicle as part of the en bloc oncologic excision. (**d**) Operative field after complete removal of the specimen, showing the resulting manubrial and costoclavicular defect. (**e**) Placement of the STRATOS titanium bar, contoured and secured between the left clavicular stump and the right second rib using rib clips to restore clavicular–sternal continuity. (**f**) Application of a Vicryl mesh to cover the anterior chest-wall defect, anchored to the rib cage and reinforced by fixation to the pectoralis major muscle.

Under general anesthesia with left lung exclusion via orotracheal intubation, the patient was placed in the supine position. A diamond-shaped incision was made at the jugular notch, followed by dissection through the subcutaneous and muscular layers to expose the pectoralis major. Bilateral exposure of the sternum and costal grid up to the clavicles was achieved. The tumor was identified, involving the left hemimanubrium, medial clavicle, and first rib. The left internal mammary pedicle was ligated using double titanium clips and a vascular loop. A partial manubriectomy was performed using a sternal saw, maintaining a 2 cm oncologic margin. The first rib and clavicle were transected with a Gigli saw, and the specimen, including skin, subcutaneous tissue, and muscle, was excised en bloc and sent for histopathologic examination. This step represented a critical technical challenge, as wide oncologic clearance had to be balanced against the preservation of sufficient clavicular and sternal bone stock to allow subsequent stable reconstruction while operating in close proximity to major mediastinal vascular structures. Hemostasis was then meticulously secured. The resection margins were verified intraoperatively through frozen-section sampling when feasible to ensure oncologic completeness without compromising reconstructive options (Figure 2).

2. Reconstruction and Stabilization

The reconstruction of the anterior chest-wall and the stabilization of the left clavicle were achieved using STRATOS. Two titanium rib clips were positioned: one on the left clavicular stump (titanium rib clip, right, 22.5°, standard 19 mm with a rotatable connector) and another (titanium rib clip, right, 45°, standard 19 mm with rotatable connector) on the right second rib along the midclavicular line. A 230 mm STRATOS titanium bar cut to 4 cm and manually contoured to reproduce the clavicular curvature was interposed between the clips and locked in place (Figure 2). Intraoperatively, we verified that the bar maintained neutral tension during shoulder manipulation, ensuring adequate mechanical stability and preventing undue stress at the clavicular stump. Bilateral pleural drains were inserted, and a Vicryl mesh was applied to cover the bony defect, anchored to the rib cage and the pectoralis major muscle. A unilateral bridging configuration was selected to replicate the natural clavicular axis while avoiding undue mediastinal traction. The final contour of the bar was refined through iterative shaping to ensure symmetric shoulder height and reduce postoperative musculoskeletal imbalance (Figure 2). Mechanically, the STRATOS bar functions as a partially constrained beam fixed at two anchorage points. According to Euler–Bernoulli beam theory, deflection *δ* under transverse load *P* is described by $\delta =\frac{PL^{3}}{EI}$. Because titanium has a high Young’s modulus (*E ≈* 110 GPa), the resulting deflection under physiologic loads is minimal, helping maintain shoulder alignment. The clips act as compression couplings that distribute forces across the cortical bone surface. For a transmitted load *F* over a contact area *A*, the local stress is $\sigma =\frac{F}{A}$, ensuring load diffusion that protects the bone and minimizes stress concentration. The bar–clip interaction thus recreates a continuous, rigid mechanical pathway between the clavicle and the thoracic skeleton.

3. Myocutaneous Flap Coverage

For soft tissue coverage, the patient was repositioned to the right lateral decubitus position. Through a left axillary incision, the thoracodorsal pedicle was identified and preserved. The latissimus dorsi muscle was harvested, detached from its humeral insertion, and rotated to the jugulo-sternal area to provide soft tissue coverage of the titanium construct. A suction drain was placed, hemostasis was verified, and closure was performed in layers using Monocryl 3-0 sutures. The patient was returned to the supine position, and the flap was secured to the thoracic wall with Vicryl 3-0 interrupted sutures. Hemostasis was confirmed, a second suction drain was placed, and the wound was closed in layers with Monocryl 3-0 and 4-0. Adequate muscle bulk provided by the rotated latissimus dorsi flap was essential to minimize the risk of implant exposure and ensure durable coverage, particularly in anticipation of postoperative adjuvant treatments. From a mechanical perspective, the soft tissue envelope provided by the latissimus dorsi flap also contributes to viscoelastic damping. Soft tissue behaves as an energy-dissipating medium characterized by a damping coefficient *c*, generating a resistive force $F_{damping}=c\cdot v$, where *v* is the relative displacement velocity between structures. This property reduces oscillatory micromotions transmitted through the titanium bar, enhancing long-term stability and decreasing the risk of irritation at the bone–implant interface.

4. Pathologic Findings

Histopathologic examination of the surgical specimen revealed a mixed neoplastic proliferation predominantly showing cartilaginous differentiation. The lesion consisted of lobules of chondrocytes with marked pleomorphism and nuclear atypia, exhibiting an expansive, infiltrative growth pattern. The mitotic count was 12 per 10 high-power fields. Foci of osteosarcomatous differentiation were also identified, characterized by malignant osteoid deposition within the cartilaginous matrix. Areas of tumor necrosis accounted for approximately 30% of the total cellularity, consistent with a partial response. Surgical resection margins were microscopically free of neoplastic infiltration. The overall morphologic features, consistent with the pre-treatment biopsy, were diagnostic of high-grade chondroblastic osteosarcoma after neoadjuvant therapy. The overlying skin and subcutaneous tissue were free of tumor involvement. These findings confirmed the tumor’s aggressive biological behavior and underscored the importance of wide-margin resection as the primary curative strategy.

5. Postoperative Course

**Figure 3:**
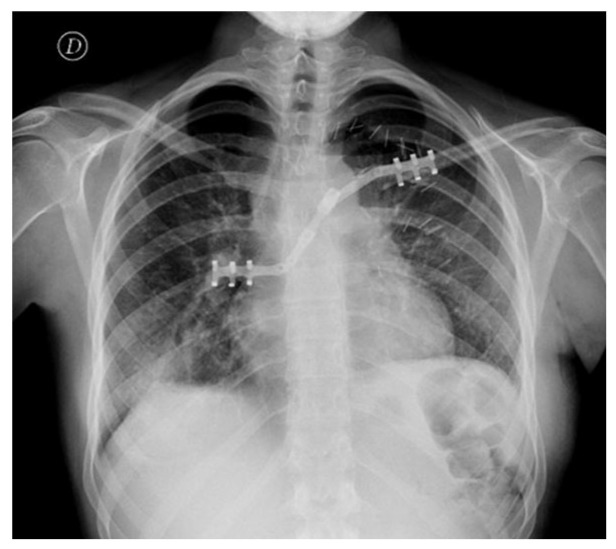
Postoperative chest X-ray showing correct alignment and fixation of STRATOS.

**Figure 4:**
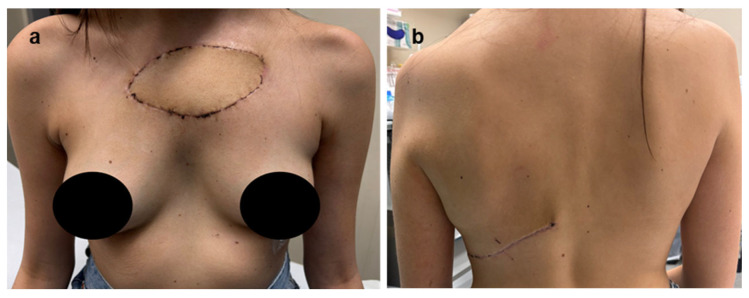
One-month postoperative clinical appearance. (**a**) Anterior view demonstrating excellent aesthetic outcome with well-healed incision and preserved thoracic contour. (**b**) Posterior view showing a well-healing latissimus dorsi flap incision with appropriate scar maturation.

The patient recovered uneventfully and was discharged on postoperative day 7 with minimal pain and preserved shoulder mobility (Figure 3). The postoperative functional outcome was excellent. One month after surgery, the patient had regained full autonomy in daily activities without assistance (Figure 4). She subsequently received adjuvant chemotherapy with ifosfamide, after which she developed grade 4 afebrile neutropenia, grade 3 thrombocytopenia, and grade 3 anemia (Common Terminology Criteria for Adverse Events, CTCAE). She then initiated mifamurtide therapy, administered twice weekly for the first 12 weeks, followed by 48 weekly cycles. At the 6-month follow-up, with no local recurrence, stable hardware positioning and no implant migration, the patient demonstrated an almost complete range of motion, with only a minimal, functionally acceptable reduction in muscular strength. Range-of-motion testing demonstrated 170° of forward flexion, 160° of abduction, and preserved internal and external rotation, indicating near-complete recovery of shoulder mechanics. No winging, instability, or hardware irritation was observed on clinical evaluation. Objective functional assessment at 6 months further supported the favorable outcome, with a Constant–Murley score of 88 points and a Disabilities of the Arm, Shoulder and Hand (DASH) score of 7.5, indicating excellent shoulder function with minimal residual disability.

## 3. Literature Review

Primary chondrosarcoma of the sternum remains rare, with most lesions involving the manubrium [6,7,8,9]. Complete en bloc resection with negative margins is the cornerstone of curative treatment, as these tumors are resistant to both chemotherapy and radiotherapy [4,5]. Various reconstructive strategies have been proposed to restore the integrity and stability of the anterior chest-wall following sternal or sternoclavicular resection. These include autologous bone grafts, methyl methacrylate–mesh composites, titanium plates, and alloplastic prostheses [10,11] (Table 1).

The STRATOS (Strasbourg Thoracic Osteosynthesis System) device, composed of modular titanium bars and clips, was initially developed for chest-wall stabilization after trauma or tumor resection [10,12]. Its biomechanical versatility and contour adaptability allow for the reconstruction of complex defects, yet its application in clavicular fixation following sternoclavicular joint (SCJ) resection has been scarcely reported.

Hajjar et al. (2013) described a 52-year-old woman with papillary thyroid carcinoma invading the manubrium, who underwent bilateral clavicular stabilization using crossed STRATOS bars anchored to the second ribs [16]. The technique achieved an excellent functional recovery, with a near-complete shoulder range of motion two months postoperatively. Similarly, Gonfiotti et al. [9] and Dingemann et al. [13] demonstrated the effectiveness of STRATOS for total sternal replacement and oncologic chest-wall repair, highlighting its rigid fixation and adaptability to various thoracic defects. However, beyond these isolated reports, there remains limited documentation of STRATOS use for reconstructing the clavicular–sternal junction, particularly after resection for primary bone malignancies such as chondrosarcoma. Despite the promising evidence, no standardized reconstructive algorithm currently exists for SCJ-involving oncologic resections, and published techniques vary considerably in terms of biomechanical reliability. The modularity and contouring capabilities of STRATOS devices may fill a significant gap in reconstructive options, particularly for young patients with high long-term functional expectations. Mechanical failure in plate- or prosthesis-based reconstructions is often driven by cyclic fatigue phenomena. Fatigue behavior follows the general relation $\sigma_{max}<\sigma_{fatigue}(N)$, where $\sigma_{max}$ is the maximum applied stress and $\sigma_{fatigue}(N)$ is the endurance limit at a given number of loading cycles. During daily activities, the clavicle undergoes hundreds to thousands of repetitive cycles of bending and torsion, making the fatigue characteristics of the reconstructive material critical. Titanium’s favorable fatigue curve, therefore, provides a theoretical advantage in maintaining long-term construct integrity in high-demand patients.

From a clinical standpoint, the adoption of the reconstructive techniques summarized in Table 1 remains heterogeneous and largely driven by institutional experience rather than standardized guidelines. Autologous grafts and methyl methacrylate–mesh constructs are still widely used due to their availability and familiarity, despite their biomechanical limitations. Conversely, titanium-based modular systems, although supported by growing evidence, are currently adopted mainly in high-volume centers with specific expertise in complex chest-wall reconstruction. This variability highlights the need for reproducible and mechanically reliable solutions, particularly in young and active patients, in whom long-term functional durability is a critical endpoint.

Recent publications have further explored alternative reconstructive strategies for sternoclavicular joint (SCJ) defects following oncologic resection, reflecting the lack of a universally accepted standard [15]. Contemporary reports describe the use of tendon autografts or allografts in figure-of-eight configurations, plate-based fixation systems, and hybrid constructs combining rigid osteosynthesis with soft tissue stabilization to restore shoulder girdle continuity. These techniques aim to balance mechanical stability with the preservation of physiological shoulder motion, although outcomes remain heterogeneous and largely derived from small case series or extrapolated from traumatic SCJ reconstructions. Reported limitations include insufficient resistance to multidirectional loading, the risk of progressive loosening, and variable durability under long-term cyclic stress. Consequently, the comparison among reconstructive options, summarized in Table 1, highlights substantial differences in biomechanical behavior, technical complexity, and suitability for young, high-demand patients undergoing extensive oncologic resections, underscoring the need for more robust and reproducible fixation strategies.

## 4. Discussion

Manubrial chondrosarcomas pose complex challenges in both oncologic and reconstructive management due to their proximity to major mediastinal vessels and the functional role of sternoclavicular articulation. Achieving an oncologically sound resection while preserving shoulder girdle stability and respiratory mechanics is essential for favorable outcomes.

Traditional methods for reconstructing the sternoclavicular region, including tendon grafts, steel wires, locking plates, or synthetic meshes, often fail to restore adequate stability and can result in chronic pain, restricted motion, or hardware failure [9,15]. Given these limitations, an alternative reconstructive strategy capable of providing both rigidity and anatomical fidelity becomes essential. STRATOS offers specific advantages in this context. From a reconstructive standpoint, the use of STRATOS for clavicular stabilization offers several biomechanical and clinical benefits. First, its anatomic adaptability allows the titanium bar to be contoured precisely to replicate the native curvature of the clavicle, thereby restoring shoulder alignment and cosmetic symmetry. Second, the system provides rigid fixation between the clavicle and the residual sternum or contralateral costal arch, preventing mediastinal displacement or shoulder girdle instability. Third, the bone-sparing design of the clips, anchoring to the cortical surface without the need for screws, reduces the risk of iatrogenic fracture, particularly in the setting of post-chemotherapy bone fragility. Finally, the low-profile, MRI-compatible titanium construction minimizes soft tissue irritation and enables safe postoperative imaging and long-term oncologic surveillance. Preoperative patient selection plays a critical role in determining the optimal reconstructive strategy. Candidates most likely to benefit from STRATOS-based clavicular stabilization include patients requiring en bloc SCJ resection with a loss of medial clavicular support, particularly young and active individuals with high functional demands. Adequate residual bone stock at the clavicular stump and at the sternal or costal anchoring site is essential to ensure secure clip fixation. Conversely, patients with extensive osteolysis, severe osteoporosis, or insufficient cortical thickness may be less suitable candidates for this technique and may require alternative reconstructive approaches. Despite these benefits, several limitations must be acknowledged. The sternoclavicular junction is a high-stress articulation, and the long-term behavior of STRATOS under repetitive mechanical loading remains uncertain. Potential risks of clip or bar fatigue fracture warrant periodic radiologic follow-up.

Furthermore, successful fixation depends on sufficient residual bone stock, both laterally on the clavicular stump and medially on the sternal or costal attachment site, to ensure stable clip anchorage. Future biomechanical testing and multicenter clinical experience will be essential to validate the durability and reproducibility of this approach in oncologic and reconstructive thoracic surgery. From a biomechanical standpoint, the clavicle functions as a strut that positions the scapula and transmits axial loads to the axial skeleton. Traditional fixation methods often fail because they do not recreate the SCJ’s tension-bearing function. The STRATOS bar, due to its rigidity and customizable curvature, more closely approximates the native biomechanical environment, thereby supporting physiologic scapulothoracic motion. The reconstructed clavicular–sternal complex behaves mechanically as a cantilever subjected to combined bending, torsional, and shear loads. Torsional moments may be expressed as $T=\frac{GJ\vartheta }{L}$, where *G* is the shear modulus of titanium, *J* is the polar moment of inertia, ϑ is the angle of rotation, and *L* is the anchored length. Axial compression generated during shoulder loading follows Hooke’s law $\Delta L=\frac{FL}{AE}$, where the minimal elongation of titanium ensures the preservation of construct length. Because the applied stresses remain well within titanium’s linear elastic region, the STRATOS bar is expected to maintain structural integrity under normal physiological conditions.

STRATOS offers distinct biomechanical advantages over traditional fixation methods for medial clavicular stabilization after sternoclavicular joint resection. From a mechanical perspective, the clavicle functions as a load-transmitting strut between the upper limb and the axial skeleton, subjected to combined bending, torsional, and shear forces during shoulder motion. Conventional techniques such as tendon grafts, cerclage wires, or standard plates often fail to reproduce this complex load-bearing role, either because they provide insufficient rigidity, are prone to elongation or fatigue, or create stress concentrations at screw–bone interfaces that may be predisposed to loosening or fracture.

In contrast, STRATOS behaves as a rigid, contoured beam that more closely approximates the native clavicular load pathway. The titanium bar can be shaped to replicate the physiological curvature of the clavicle, thereby restoring normal moment arm and reducing abnormal shear forces at the clavicular stump. Its high flexural rigidity, derived from the elastic modulus of titanium and the bar’s cross-sectional geometry, limits deformation under physiological loads and helps maintain shoulder alignment during dynamic activities. Moreover, the clip-based anchorage distributes forces over a broader cortical surface without penetrating the bone, minimizing stress risers and reducing the risk of iatrogenic fracture, particularly in young patients or in bone weakened by chemotherapy.

Compared with plate-and-screw constructs, which primarily resist bending in a single plane, the STRATOS bar–clip interface provides multidirectional stability and improved resistance to torsional moments generated during scapulothoracic motion. This configuration also reduces micromotion at the bone–implant interface, a key factor in the long-term durability of the construct under cyclic loading. Collectively, these biomechanical properties allow STRATOS to restore clavicular–sternal continuity in a manner that is both rigid and anatomically faithful, offering a mechanical advantage over traditional fixation methods in this highly demanding reconstructive setting.

In the present case, the use of a contoured STRATOS bar to bridge the left clavicular stump and the contralateral costal margin provided excellent mechanical stability and functional restoration. The cantilever-like configuration of the clavicle requires constructs that resist axial rotation and superior–inferior shear, both of which are adequately counteracted by the rigid STRATOS bar–clip interface. Long-term cyclic loading remains the dominant mechanical risk for any clavicular reconstruction. Fatigue life can be estimated using Basquin’s law, $\sigma_{\alpha }=\sigma_{f}^{\prime }{\left(2F\right)}^{b}$, where $\sigma_{\alpha }$ represents stress amplitude, $\sigma_{f}^{\prime }$ the fatigue strength coefficient, *b* the fatigue exponent, and *N* the number of load cycles to failure. Given that shoulder motion may exceed one million cycles annually, titanium’s high endurance limit significantly mitigates the risk of structural fatigue.

Nevertheless, periodic radiologic supervision is warranted, particularly in younger and more active patients. The system’s capacity to replicate physiological shoulder alignment proved critical in preserving motion and cosmesis. In addition to skeletal reconstruction, soft tissue coverage is a key determinant of postoperative outcomes. Various myocutaneous flaps have been described in the literature for anterior chest-wall reconstruction, including the pectoralis major, rectus abdominis, latissimus dorsi, and omental flaps. These techniques provide robust vascularized coverage, reduce the risk of infection, and contribute to improved contour restoration. The choice of flap should be individualized based on defect size, prior surgeries, and patient-specific factors, underscoring the importance of a multidisciplinary approach involving plastic surgeons early in the treatment planning process. This mirrors the findings of Hajjar et al. [16], who reported successful clavicular reconstruction after manubrial resection for thyroid metastasis, with excellent recovery of range of motion and strength. Together, these cases demonstrate that STRATOS-based stabilization can effectively maintain shoulder girdle integrity after oncologic SCJ resection, expanding its role beyond traditional chest-wall reconstruction.

At six months, our patient exhibited a full pain-free range of motion, stable fixation, and no radiologic evidence of hardware displacement or local tumor recurrence. These results confirm the feasibility of adapting STRATOS for complex clavicular reconstructions requiring both rigidity and anatomical precision. Beyond the mechanical performance of the construct, comprehensive reconstruction must also address the soft tissue and aesthetic implications of anterior chest-wall surgery, for which the involvement of a plastic surgery team is a critical component.

Beyond ensuring adequate soft tissue coverage of the prosthetic construct, plastic reconstruction plays a central role in restoring thoracic contour and minimizing visible deformity. Given the significant psychological impact associated with chest-wall defects, particularly in young women, achieving an acceptable aesthetic outcome contributes substantially to postoperative body image, emotional well-being, and overall quality of life. Early integration of reconstructive planning within the multidisciplinary strategy is therefore essential to optimize both functional and psychosocial outcomes. This case supports the role of modular titanium systems as a valuable option when conventional clavicular reconstruction techniques cannot provide sufficient stability. Given the limited available reports, this technique should currently be considered in highly selected cases, particularly when autologous or plate-based options cannot guarantee adequate stability. The accumulation of multicenter experience will be essential for defining indications, long-term outcomes, and potential modifications to the technique.

Despite the favorable early clinical and functional results observed in this case, several potential long-term complications specific to this novel application of STRATOS must be acknowledged. These include fatigue-related bar or clip failure under prolonged cyclic shoulder loading, progressive bone remodeling or cortical thinning at the clip–bone interface, and late-onset alterations in shoulder girdle biomechanics due to the rigid nature of the reconstruction. In addition, the long-term interaction between the titanium construct and surrounding soft tissues, particularly in young and active patients, remains incompletely characterized. Extended follow-up and the accumulation of multicenter experience will therefore be essential to assess durability, identify late complications, and refine patient selection criteria for this reconstructive approach.

## 5. Conclusions

This case illustrates a novel and practical application of STRATOS for medial clavicular stabilization following SCJ resection for manubrial chondrosarcoma. The system provided durable fixation, excellent functional and aesthetic outcomes, and early recovery without complications. Compared with conventional techniques, STRATOS offers superior adaptability and biomechanical stability. While this single case cannot establish definitive guidelines, it highlights the potential of STRATOS bars to evolve into a standardized reconstructive option in oncologic thoracic surgery. This case suggests that integrating principles of beam mechanics, load transmission, and material fatigue into thoracic reconstruction is essential to replicate physiologic clavicular biomechanics and ensure durable functional outcomes.

## Figures and Tables

**Table 1:** Comparison of reconstruction options after sternoclavicular joint (SCJ) resection.

Reconstruction Method	Biomechanical Stability	Advantages	Limitations/Risks	When It Is Most Useful
Autologous tendon or fascia lata grafts	Low–moderate	Biological material; no hardware; good infection profile	Limited rigidity; elongation over time; technically demanding	Small defects; low-demand patients; infection-prone fields
Locking plates/clavicular plates	Moderate	Familiar orthopedic technique; immediate rigidity	Prominent hardware; screw pull-out; risk of fracture in osteopenic bone; contour mismatch to sternum	Traumatic SCJ instability; adequate bone stock
Titanium plates or meshes	Moderate–high	Easily shaped; stable fixation; widely available	Hardware fatigue; risk of deformation; may interfere with imaging	Moderate-size manubrial defects; need for rigid anterior support
Alloplastic sternal or manubrial prostheses	High (for central support)	Good structural replacement; customizable	Bulky; may not restore clavicular–sternal biomechanics; requires large exposure	Large manubrial defects without clavicular involvement
STRATOS bar-and-clip system (current case)	High (rigid, anatomically contoured)	Bone-sparing clip design; customizable bar contour; strong clavicular–sternal continuity; MRI-compatible	Long-term fatigue resistance unknown; requires adequate bone for clip anchorage	Complex oncologic resections requiring both stability and reconstruction of clavicular alignment

## Data Availability

The original contributions presented in this study are included in the article. Further inquiries can be directed to the corresponding author.
